# Comparison of Retinal and Choroidal OCT Measures and Features at Term Equivalent Age in Preterm and Term Infants

**DOI:** 10.1167/iovs.66.13.40

**Published:** 2025-10-27

**Authors:** Seong Joon Ahn, Jocelyn He, Vincent Tai, Katrina P. Winter, Neeru Sarin, Shwetha Mangalesh, Xi Chen, Gui-Shuang Ying, Cynthia A. Toth

**Affiliations:** 1 Department of Ophthalmology, Duke University School of Medicine, Durham, North Carolina, United States; 2 Department of Ophthalmology, Perelman School of Medicine, University of Pennsylvania, Philadelphia, Pennsylvania, United States

**Keywords:** choroid, optical coherence tomography, foveal development, retinopathy of prematurity, preterm birth

## Abstract

**Purpose:**

To evaluate macular optical coherence tomography (OCT) features at term-equivalent age in preterm and term infants, and to test the hypothesis that both preterm birth and retinopathy of prematurity (ROP) are associated with differences in OCT measures and features at term-equivalent age, with the choroid potentially influencing the outer retina.

**Methods:**

This study analyzed data for 162 preterm and 78 term infants from prospective, handheld OCT imaging studies, with images of both eyes at 37 to 42 weeks postmenstrual age (PMA). Quantitative and qualitative features, from reading center grading, were compared between preterm and term infants, with subgroup comparison between stage <2 vs. more severe ROP. Multivariate analyses explored the associations of OCT features with variables including gestational age (GA), ROP, and choroidal thickness, adjusting for PMA at imaging and intereye correlation using generalized estimating equations.

**Results:**

Compared to term infants, preterm infants had a greater total and inner retinal thickness at the fovea (232.4 [mean] ± 126.9 [SD] vs. 108.0 ± 35.3 µm and 47.3 ± 20.7 vs. 25.0 ± 14.8 µm, respectively; both *P* < 0.001), and thinner choroid (267.2 ± 90.3 vs. 350.2 ± 161.4 µm; *P* < 0.001). Rates of intraretinal fluid were higher (63.8 vs. 6.6%; *P* < 0.001) in preterm infants, whereas subretinal fluid was only present (7.0%) in term infants. Despite similar outer retinal thickness, the ellipsoid zone was more frequently observed at the foveal center in term infants (35.2%) than preterm infants (8.5%; *P* < 0.001). Multivariate analyses identified significant associations between higher GA and reduced foveal inner and outer retinal thicknesses. More severe ROP and decreased choroidal thickness were associated with increased foveal inner retinal thickness (both *P* < 0.01), but not with outer retinal thickness.

**Conclusions:**

By term-equivalent age, foveal inner retinal immaturity and photoreceptor under-development is evident in preterm versus term-born retinas. A thinner choroid is associated with thicker inner retina.

Preterm birth, defined as birth before 37 weeks of gestation, affects approximately 10% of all births worldwide and is associated with various developmental challenges,^1^ including those related to visual function and retinal development.^2^^,^^3^ The retina, a complex neural tissue responsible for initiating the visual process, undergoes maturation during the third trimester of pregnancy and early postnatal period. Premature birth interrupts this crucial developmental phase, potentially leading to structural and functional alterations in the retina that persist beyond infancy.^3^

Handheld optical coherence tomography (OCT) has emerged as a valuable, noninvasive imaging modality to assess retinal microstructural alterations.^4^^,^^5^ This technology provides high-resolution, cross-sectional images of the retina, allowing for detailed examination of retinal layers and the choroid in neonates.^6^^,^^7^ Its application in neonatal care has expanded our understanding of retinal development in both preterm and term infants, offering insights into the potential impact of prematurity on ocular structures.^3^ It is useful for examining retinal pathologies and less stressful than traditional binocular indirect ophthalmoscopy.^8^^,^^9^ Furthermore, it is a reliable tool for measuring retinal and choroidal thickness, providing reproducible measurements comparable to those obtained with tabletop OCT instruments.^10^^,^^11^

Previous studies with OCT have reported various findings in preterm infants, including altered foveal morphology, delayed photoreceptor development, and the presence of cystoid macular edema.^8^^,^^12^^,^^13^ However, most of these studies focused on specific aspects of retinal structure or limited their analysis to particular gestational age groups.^8^^,^^13^ A comprehensive comparison of OCT measures and features between preterm and term infants at term-equivalent age is still needed, particularly to differentiate the potential influence of prematurity and prematurity-associated conditions, such as retinopathy of prematurity (ROP), on retinal development at this age.

Among the potential factors associated with retinal development, the choroid may play a role in retinal development by supplying oxygen and essential nutrients, such as glucose, to the outer retina.^14^ Recent evidence suggests that choroidal thickness may be altered in preterm infants with prematurity-associated conditions^10^ and potentially in term infants,^14^ although conflicting results exist regarding the association between the choroid and preterm birth or ROP.^15^ However, the relationship between choroidal changes and retinal development in the context of prematurity and ROP remains poorly understood.

This study aims to investigate retinal and choroidal development by comparing OCT measures and features at term-equivalent age between preterm infants with varying severity of ROP and term infants. Through secondary analyses of a large infant retinal OCT database from multiple previous studies, we explore the relationships between retinal and choroidal quantitative and qualitative features in the context of prematurity and ROP. We hypothesize that preterm infants exhibit different OCT measures compared to term infants, reflecting variations in retinal development and maturation. Additionally, we hypothesize that ROP may further impact these OCT features and measures and that choroidal thickness is associated with outer retinal development.

## Methods

### Study Design and Participants

This observational, cross-sectional study was conducted from the secondary analyses of multiple OCT databases from previous Duke Health Institutional Review Board–approved prospective studies including (1) BabySTEPS1 (R01EY025009; enrolled between August 12, 2016 and December 30, 2020, *n* = 82 infants), (2) Healthy term infant arm of Bedside Optical Retinal Assessment of Hypoxic Ischemic Encephalopathy in Infants (R21EY029384; enrolled from August 2015 to June 2017, *n* = 28), (3) association of inflammatory markers and cystoid macular edema in very preterm infants study (Research to Prevent Blindness grant; enrolled between July 1, 2015 and December 21, 2015, *n* = 8), (4) OCT at bedside study (a pilot Duke Clinical and Translational Science Project Award; enrolled between July 30, 2009 and March 25, 2016, *n* = 4), and (5) spectral-domain OCT imaging of infant eyes study (Hartwell Foundation research grant; enrolled between January 15, 2009, to July 20, 2009, *n* = 122).^8^^,^^16^^–^^19^ All of these prospective studies had obtained informed parental or guardian consent prior to the research OCT imaging. Data from preterm infants born before 37 weeks of gestation and term infants born between 37 and 42 weeks of gestation were included in this study, with OCT images obtained for both groups at a postmenstrual age (PMA) between 37 and 42 weeks. Infants or eyes were excluded if they had significant congenital anomalies or ocular pathologies unrelated to prematurity, or if the OCT images were of unacceptable quality for image analysis (*n* = 33 eyes [18 from preterm and 15 from term infants]; 26 of these were excluded because of insufficient choroidal imaging). None of the stage 4 ROP patients (all classified as stage 4a) had involvement of the macular area or undergone vitrectomy or any major surgical intervention prior to the OCT assessment, ensuring that the OCT measurements accurately reflected retinal morphology without potential bias from retinal detachment or surgical interventions. This study adhered to the tenets of the Declaration of Helsinki. The Duke Health Institutional Review Board determined that this secondary analysis study met the necessary privacy guidelines and the Food and Drug Administration regulations and thus was exempt from further review.

### OCT Imaging and Evaluations

OCT imaging had been performed by multiple trained operators using one of three handheld OCT systems: the Leica Envisu spectral-domain (SD) OCT (used in Studies 1, 3, 4, and 5), two investigational swept-source (SS) OCT systems, and the ultracompact (UC) 2 (100 kHz, Study 1) and UC3 (200 kHz, Studies 1 and 2). These systems demonstrated excellent repeatability and reproducibility of axial and lateral measurements, comparable to those of commercially available SD-OCT devices.^20^ In this study, PMA was recorded as both weeks and days, and we analyzed images from a single OCT imaging session, obtained between 37 and 42 weeks PMA in the eyes of both preterm and term infants. When multiple visits occurred within this window, the visit closest to 39 weeks PMA was used, provided that the image quality was excellent or acceptable, according to the grading scheme presented in previous studies.^8^ If the image quality at the closest visit was unacceptable, the visit with the best quality image within the 37- to 42-week period was selected. The PMA at imaging was calculated using the best estimate of gestational age from the infant's medical records.

For qualitative OCT features, two experienced OCT graders (KPW and SJA), blinded to clinical information, independently identified all pathological features, including intraretinal fluid (IRF) and subfoveal subretinal fluid (SRF), in the macular volume scan from each imaging session. Features of outer retinal development, such as the presence of an ellipsoid zone (EZ) band and external limiting membrane (ELM) at the foveal center, were also evaluated by the graders from the best foveal scan. Any discrepancies were resolved through consensus, in consultation with a senior investigator (CAT).

For quantitative analyses, the retinal layers were semi-automatically segmented using the proprietary infant-specific DOCTRAP software, with manual corrections of segmentation errors if necessary. Two independent, experienced graders (KPW and SJA) evaluated the automated segmentations, and manually corrected segmentations by one grader were also reviewed by the other. OCT features were also reviewed independently by both reviewers, and any discrepancies in OCT features or segmentations were resolved through a consensus review with the senior investigator (CAT). Thickness data at the foveal center and parafovea were extracted using custom MATLAB codes for the OCT measures analyzed in our previous study,^17^ including total retinal thickness (from the internal limiting membrane [ILM] to the inner border of the retinal pigment epithelium (RPE)/Bruch's membrane complex line), inner retinal layer thickness (IRL; from the ILM to the boundary between the inner plexiform layer and inner nuclear layer [INL]), and outer retinal layer thickness (ORL; from the boundary between the INL and outer plexiform layer to Bruch's membrane) at both the fovea and (nasal and temporal) parafovea. Additionally, INL thickness and choroidal thickness were obtained by averaging over a central 2 mm across the fovea on the horizontal section. INL was separated from the IRL to minimize the effect of macular edema on IRL thickness, as this layer is most affected by the presence and severity of macular edema. The parafoveal to foveal thickness (PF) ratio was also calculated for total retinal thickness and thickness of the IRL and ORL.

Clinical data collected included gestational age, birth weight, sex, race, and ROP status and severity (maximal ROP stage), classified according to the International Classification of Retinopathy of Prematurity (ICROP).^21^ Other prematurity-associated comorbidities such as patent ductus arteriosus (PDA), sepsis, intraventricular hemorrhage (IVH), periventricular leukomalacia, bronchopulmonary dysplasia (BPD), necrotizing enterocolitis (NEC), and systemic treatments such as indomethacin, transfusion, and maternal antenatal steroids, were also recorded.

### Statistical Analyses

Descriptive analyses were performed for the demographics, clinical characteristics, and quantitative and qualitative OCT features of the included infants using mean, standard deviation (SD) for continuous measures, and using count and percentage for categorical measures. Comparisons of demographics and clinical characteristics between preterm and term infants were performed using independent t-tests or Mann–Whitney U tests for continuous measures; and chi-square or Fisher's exact tests for categorical measures. Comparisons of OCT features between preterm and term infants were performed using generalized regression models that account for the inter-eye correlation using generalized estimating equations (GEE). Preterm infants were further subdivided into those with minimal (stage <2) ROP and those with stage ≥2 ROP, and comparisons between these two subgroups were performed similarly as comparisons between term and pre-term infants. Multivariate regression models were employed to identify the factors associated with inner and outer retinal thicknesses, and to explore the relationships between choroidal and retinal thicknesses (inner and outer) by adjusting for PMA at imaging and inter-eye correlation. Univariate and multivariate logistic regression models that accounted for inter-eye correlation using GEE were performed to determine factors associated with the presence of EZ band at the fovea at term-equivalent age, the odds ratios (ORs) and 95% confidence intervals (95% CIs) were calculated for each risk factor. All statistical analyses were performed using SAS version 9.4 (SAS Institute Inc., Cary, NC, USA) and two-sided P < 0.05 was considered statistical significant.

## Results

### Demographic and Clinical Characteristics of the Study Population

Table 1 presents a comparison of demographic and clinical characteristics between preterm infants (*N* = 162) and term infants (*N* = 78). The mean gestational age was 26.7 weeks in preterm infants and 38.9 weeks in term infants. The PMA at imaging was similar (mean 39.3 weeks in both groups) between the groups (*P* = 0.91). While gender distribution was comparable (*P* = 0.52), racial distribution showed significant difference, a higher percentage of African-American (52.5%) preterm infants compared to term infants (38.5%; *P* < 0.001). With the exception of periventricular leukomalacia (*P* = 0.18), preterm infants had significantly higher rates of systemic comorbidities, such as PDA, sepsis, IVH, BPD, and NEC (all *P* < 0.01). Preterm infants showed varying severities of ROP, with 64.5% of preterm eyes reaching a maximum ROP stage of ≥2.

**Table 1.** Comparison of Demographic and Clinical Characteristics Between the Preterm and Term Infants

					*P* Value	
Characteristics	Group 1 Term (*N* = 78)	Group 2 Preterm (*N* = 162)	Group 2-1 Preterm With Minimal (Stage <2) ROP (*N* = 58)	Group 2-2 Preterm With More Severe ROP (*N* = 104)	Group 1 vs. 2	Group 2-1 vs. 2-2
Gestational age (wks)					**<0.001**	**<0.001**
Mean (SD)	38.9 (1.0)	26.7 (2.7)	29.1 (2.0)	25.4 (2.0)		
Range	37.0–40.0	23.0–34.6	25.0–34.6	23.0–34.4		
Birth weight (g)					**<0.001**	**<0.001**
Mean (SD)	3314 (423)	875 (266)	1099 (220)	750 (199)		
Range	2435.0–4395.0	420.0–1535.0	660–1494	420–1535		
PMA at imaging (wks)					0.91	0.52
Mean (SD)	39.33 (0.95)	39.32 (0.99)	39.25 (1.19)	39.36 (0.87)		
Range	37.29–41.14	36.86–42.29	36.86–42.29	36.86–41.00		
Gender					0.52	0.18
Female	41 (52.6%)	78 (48.1%)	32 (55.2%)	46 (44.2%)		
Male	37 (47.4%)	84 (51.9%)	26 (44.8%)	58 (55.8%)		
Race					**<0.001**	0.11
African-American/More than one	30 (38.5%)	85 (52.5%)	29 (50.0%)	56 (53.8%)		
White	30 (38.5%)	72 (44.4%)	25 (43.1%)	47 (45.2%)		
Others/unknown	18 (23.1%)	5 (3.1%)	4 (6.9%)	1 (1.0%)		
Comorbidities/systemic treatment			
Patent ductus arteriosus	0 (0%)	86 (53.1%)	22 (37.9%)	64 (61.5%)	**<0.001**	**0.004**
Sepsis	0 (0%)	108 (66.7%)	26 (44.8%)	82 (78.8%)	**<0.001**	**<0.001**
Intraventricular hemorrhage	0 (0%)	60 (37.0%)	11 (19.0%)	49 (47.1%)	**<0.001**	**<0.001**
Periventricular leukomalacia	0 (0%)	5 (3.1%)	0 (0.0%)	5 (4.8%)	0.18	0.16^*^
Bronchopulmonary dysplasia	0 (0%)	22 (13.6%)	2 (3.4%)	20 (19.2%)	**<0.001**	**0.005**
Necrotizing enterocolitis	0 (0%)	14 (8.6%)	2 (3.4%)	12 (11.5%)	**0.006**	0.08
Treatment with indomethacin	0 (0%)	83 (51.2%)	13 (22.4%)	70 (67.3%)	**<0.001**	**<0.001**
Transfusion	0 (0%)	140 (86.4%)	41 (70.7%)	99 (95.2%)	**<0.001**	**<0.001**
Maternal antenatal steroids	0 (0%)	143 (88.3%)	50 (86.2%)	93 (89.4%)	**<0.001**	0.54
Ocular conditions, eyes		307	109	198		
Maximal stage						
0	N/A	77 (25%)	77 (70.6%)		N/A	
1	N/A	32 (10%)	32 (29.4%)		N/A	
2	N/A	114 (37%)		114 (57.6%)	N/A	
3	N/A	73 (24%)		73 (36.9%)	N/A	
4	N/A	1 (0%)		1 (0.5%)	N/A	
4A	N/A	8 (3%)		8 (4.0%)	N/A	
4B	N/A	2 (1%)		2 (1.0%)	N/A	
Plus disease						
None	N/A	191 (62%)	109 (100.0%)	82 (41.4%)	N/A	
Pre-plus	N/A	52 (17%)		52 (26.3%)	N/A	
Plus	N/A	64 (21%)		64 (32.3%)	N/A	
Treatment						
None	N/A	220 (72%)	109 (100.0%)	111 (56.1%)	N/A	
Both	N/A	23 (8%)		23 (11.6%)	N/A	
Laser	N/A	50 (16%)		50 (25.3%)	N/A	
Avastin	N/A	14 (5%)		14 (7.1%)	N/A	

Boldface indicates statistical significance.

* Fisher's exact test.

Subgroup comparisons between preterm infants with minimal (Stage <2) ROP and those with more severe (≥2) ROP revealed several differences in demographic and clinical characteristics. Preterm infants with more severe ROP had a significantly lower mean gestational age and birth weight compared to those with minimal ROP (both *P* < 0.001), and more systemic comorbidities such as PDA, sepsis, IVH, BPD, and NEC; and systemic treatment such as indomethacin and transfusion (all *P* < 0.05). PMA at imaging, gender, and race distribution were comparable between the two groups. Some eyes in the group with more severe ROP exhibited pre-plus (26.3%) and plus disease (32.3%) and underwent treatment interventions (43.9%), including laser therapy (25.3%), Avastin injections (7.1%), or a combination of both (11.6%), during their clinical course.

### Comparison of Qualitative OCT Features Among Groups

Table 2 presents a comparison of qualitative OCT features among term infants, preterm infants, and preterm subgroups with minimal and more severe ROP. IRF (Supplementary Fig. S1) was significantly more frequent in preterm infants (63.8%) compared to term infants (6.6%; *P* < 0.001), with a higher prevalence in preterm infants with more severe ROP (69.2%) than those with minimal ROP (54.1%), although the difference was statistically insignificant. SRF (Supplementary Fig. S1) was observed in a small proportion of term infants (7.0%) but was absent in all preterm groups (*P* < 0.001).

**Table 2.** Comparison of Qualitative Features on OCT in Preterm and Term Infants, Including Subgroups of Preterm Infants With Minimal and More Severe ROP

					*P* Values		
Feature	Group 1 Term (*N* = 143)	Group 2 Preterm (*N* = 307)	Group 2-1 Preterm With Minimal ROP (*N* = 109)	Group 2-2 Preterm With More Severe ROP (*N* = 198)	1 vs. 2	1 vs. 2-1	2-1 vs. 2-2
Intraretinal fluid	9/137 (6.6%)	196/307 (63.8%)	59/109 (54.1%)	137/198 (69.2%)	**<0.001**	**<0.001**	0.06
Subretinal fluid	10/143 (7.0%)	0/307 (0.0%)	0/109 (0.0%)	0/198 (0.0%)	**<0.001** ^*^	**<0.001** ^*^	1.0^*^
ELM at foveal center	59/128 (46.1%)	47/299 (15.7%)	20/107 (18.7%)	27/192 (14.1%)	**<0.001**	**<0.001**	0.40
EZ Band at foveal center	50/142 (35.2%)	26/306 (8.5%)	9/109 (8.3%)	17/198 (8.6%)	**<0.001**	**<0.001**	0.93

N/A, not applicable GEE is used for accounting inter-eye correlation.

Boldface indicates statistical significance.

* Fisher's exact test was used because the P-value could not be calculated using GEE due to a 0% occurrence of the feature in one of the comparison groups.

The microstructural parameters of outer retinal development also differed significantly between the groups (Table 2). The presence of the ELM and EZ bands at the foveal center was less common in preterm infants (ELM: 15.7%, EZ: 8.5%) compared to term infants (ELM: 46.1%, EZ: 35.2%; both *P* < 0.001). Subgroup comparisons revealed that there were no statistically significant differences between preterm infants with minimal (ELM: 18.7%, EZ: 8.3%) and more severe ROP (ELM: 14.1%, EZ: 8.6%; both *P* > 0.05). However, there were statistically significant differences between term infants and preterm infants with minimal ROP (both *P* < 0.001).

### Comparison of Quantitative OCT Features Among Groups

Quantitative OCT measures between preterm and term infants, focusing on retinal layer and choroidal thickness, are presented in Table 3. The mean total retinal thickness at the fovea was significantly greater in preterm infants (232.4 ± 126.9 [SD] µm) compared to term infants (110.4 ± 40.6 µm; *P* < 0.001). This difference persisted in the IRL thickness at the fovea, with preterm infants exhibiting an average thickness of 47.3 ± 20.7 µm compared to 25.0 ± 14.8 µm in term infants (*P* < 0.001), whereas there was no significant difference in ORL thickness at the fovea between the groups. The parafoveal IRL and ORL thicknesses also differed significantly, being greater in term infants (146.7 ± 45.9 and 108.9 ± 37.3 µm, respectively) compared to preterm infants (126.5 ± 21.2 and 92.1 ± 19.8 µm, respectively; both *P* = 0.002). Notably, INL thickness across the central 2 mm was substantially higher in preterm infants (96.4 ± 69.4 µm) than in term infants (42.1 ± 21.8 µm; *P* < 0.001). However, the choroidal thickness across the central 2 mm was significantly thinner in preterm infants (267.2 ± 90.3 µm) than in term infants (350.2 ± 161.4 µm; *P* < 0.001). Comparisons between preterm infants with minimal ROP and those with more severe ROP showed significant differences in most parameters, except for total and outer retinal thicknesses at the fovea and the INL across the central 2 mm (Table 3).

**Table 3.** Comparison of Quantitative OCT Measurements and PF Thickness Ratios [Mean (SD)] Between Term and Preterm Infants, Including Subgroups of Preterm Infants With Minimal and More Severe ROP

					*P* Values^*^		
Layer/Location	Group 1 Term (*N* = 143 Eyes)	Group 2 Preterm (*N* = 307)	Group 2-1 Preterm With Minimal ROP (*N* = 109)	Group 2-2 Preterm With More Severe ROP (*N* = 198)	1 vs. 2	1 vs. 2-1	2-1 vs. 2-2
Total retina-RPE							
Fovea	110.4 (40.6)	232.4 (126.9)	225.4 (142.8)	236.3 (117.5)	**<0.001**	**<0.001**	0.62
Parafovea**^†^**	298.3 (97.6)	279.9 (42.5)	291.8 (29.1)	271.9 (48.0)	0.17	0.63	**0.003**
Inner retinal layer^‡^							
Fovea	25.0 (14.8)	47.3 (20.7)	38.5 (15.9)	52.2 (21.5)	**<0.001**	**<0.001**	**<0.001**
Parafovea**^†^**	146.7 (45.9)	126.5 (21.2)	138.0 (15.3)	118.7 (21.1)	**0.002**	0.17	**<0.001**
Outer retinal layer**^§^**							
Fovea	88.5 (34.2)	96.6 (38.4)	94.6 (41.0)	97.6 (37.0)	0.08	0.33	0.62
Parafovea**^†^**	108.9 (37.3)	92.1 (19.8)	99.2 (16.5)	87.4 (20.5)	**0.002**	0.08	**<0.001**
Inner nuclear layer across center 2 mm	42.1 (21.8)	96.4 (69.4)	95.7 (73.0)	96.9 (67.1)	**<0.001**	**<0.001**	0.93
Choroid across center 2 mm	350.2 (161.4)	267.2 (90.3)	301.7 (93.6)	246.0 (81.6)	**<0.001**	0.0503	**0.001**
PF ratio for inner retinal thickness	8.57 (10.33)	3.38 (1.84)	4.18 (1.71)	2.84 (1.73)	**<0.001**	**<0.001**	**<0.001**
PF ratio for outer retinal thickness	1.22 (0.20)	1.03 (0.28)	1.14 (0.27)	0.96 (0.25)	**<0.001**	0.08	**<0.001**
PF ratio for total thickness	2.73 (0.52)	1.60 (0.74)	1.84 (0.85)	1.43 (0.61)	**<0.001**	**<0.001**	**0.005**

Boldface indicates statistical significance.

* GEE is used for accounting inter-eye correlation.

† Average of nasal and temporal.

‡ From the ILM to the boundary between the inner plexiform layer and the INL.

§ From the boundary between the INL and outer plexiform layer to the Bruch's membrane.

The mean PF ratio of the total retina thickness was significantly higher in term infants compared to preterm infants (2.73 ± 0.52 vs. 1.60 ± 0.74; *P* < 0.001), indicating that term infants had a relatively thicker parafovea compared to fovea than in preterm infants. Additionally, the mean PF ratio for IRLs was remarkably higher in term infants compared to preterm infants (8.57 ± 10.33 vs. 3.38 ± 1.84; *P* < 0.001). The ratio for ORLs, however, was slightly higher in term infants than in preterm infants (1.22 ± 0.20 and 1.03 ± 0.28 in term and preterm infants, respectively; *P* < 0.001).

### Association of Choroidal Thickness and Clinical Variables With Retinal Thickness and Foveal EZ Band

Table 4 presents the association of clinical characteristics and OCT features with outer retinal thickness at the fovea. Univariate analysis reveals that choroidal thickness was not associated with outer retinal thickness at the fovea; however, PMA at imaging, GA at birth, and presence of foveal EZ band were significantly associated with outer retinal thickness (all *P* < 0.05). After adjusting for PMA at imaging, only GA at birth was significantly associated with outer retinal thickness at the fovea (*P* = 0.03).

**Table 4.** Association of Clinical Characteristics and OCT Features With Outer Retinal Thickness at the Fovea in Preterm and Full-Term Infants

	Outer Retinal Thickness at the Fovea				
		Univariate		Multivariate	
Variables	N (Eyes)	Regression Coefficient	*P* Value^*^	Regression Coefficient	*P* Value^*^
PMA at imaging (per week)	447	7.24 (2.17)	**0.002**		
GA at birth (per week)	447	−0.73 (0.34)	**0.036**	−0.75 (0.33)	**0.027**
Choroidal thickness (per 100 µm)	296	4.74 (2.90)	0.20	4.44 (2.91)	0.22
Gender	447		0.28		0.41
Female	224 (50.1%)	96.40 (3.48)^†^		95.86 (3.43)^‡^	
Male	223 (49.9%)	91.64 (2.74)^†^		92.19 (2.72)^‡^	
Race	447		0.69		0.75
White	191 (42.7%)	96.01 (3.04)^†^		95.70 (3.01)^‡^	
African-American/ More than one	213 (47.7%)	92.04 (3.52)^†^		92.32 (3.41)^‡^	
Others/unknown	43 (9.6%)	95.09 (6.74)^†^		95.04 (6.83)^‡^	
Foveal EZ band	445		**0.015**		0.08
No	370 (83.1%)	91.76 (2.44)^†^		92.30 (2.39)^‡^	
Yes	75 (16.9%)	103.11 (3.99)^†^		100.46 (4.11)^‡^	
ROP stage	307		0.62		0.70
<2	109 (35.5%)	94.60 (5.15)^†^		95.09 (5.08)^‡^	
≥2	198 (64.5%)	97.64 (3.23)^†^		97.37 (3.14)^‡^	
ROP treatment^§^	307		0.20		0.20
No treatment	220 (72%)	96.71 (3.33)^†^		96.85 (3.28)^‡^	
Laser photocoagulation	50 (16%)	87.89 (6.85)^†^		88.52 (6.67)^‡^	
Anti-VEGF injection	14 (5%)	99.26 (10.81)^†^		94.62 (11.35)^‡^	
Both	23 (8%)	112.34 (7.75)^†^		112.51 (7.55)^‡^	

GEE is used for accounting intereye correlation.

Boldface indicates statistical significance.

* Adjusted by PMA at imaging.

† Mean (SE).

‡ Adjusted mean (SE).

§ Treatment was after OCT session.

Supplementary Table S1 details the relationship between clinical characteristics and OCT features with inner retinal thickness at the fovea. Both univariate analysis and multivariate analysis found that choroidal thickness (*P* = 0.005), along with gestational age at birth (*P* < 0.001), race (*P* = 0.002), ROP stage (*P* < 0.001), and foveal EZ band (*P* = 0.001), were significantly associated with inner retinal thickness.

Regarding the relationship between clinical characteristics and choroidal thickness (Supplementary Table S2), higher GA at birth demonstrated a strong positive correlation with increased central choroidal thickness in both univariate and PMA-adjusted multivariate models (*P* < 0.001). Furthermore, eyes with more severe ROP exhibited significantly thinner choroids compared to those with stage <2 (*P* < 0.001), indicating that advanced ROP may be associated with impaired choroidal growth.

Logistic regression analyses were performed to identify clinical characteristics and OCT features associated with the presence of the EZ band at the fovea (Table 5). In univariate analysis, both PMA at imaging and GA showed significant associations with the presence of EZ band at the fovea (OR = 1.66; 95% CI, 1.17–2.36; *P* = 0.004 and OR = 1.14; 95% CI, 1.08–1.21; *P* < 0.001, respectively). In terms of race, univariate analysis indicated that infants categorized as others had significantly higher odds of having a foveal EZ band compared to white infants (OR = 4.84; 95% CI, 2.36–9.94; *P* = 0.005). However, choroidal thickness did not show a significant association in the univariate or multivariate analysis, as were other clinical variables (all *P* > 0.05).

**Table 5.** Association of Clinical Characteristics and OCT Features With Presence of Foveal Ellipsoid Zone in Preterm and Full-Term Infants

			Univariate		Multivariate^*^	
Variables	N (Eye)	No. of Eyes With Foveal Ellipsoid Zone (%)	OR (95% CI)	*P* Value	OR (95% CI)	*P* Value
PMA at imaging (per week)	448	76 (17%)	1.66 (1.17, 2.36)	**0.004**		
GA at birth (per week)	448	76 (17%)	1.14 (1.08, 1.21)	**<0.001**	1.14 (1.08, 1.21)	**<0.001**
Choroidal thickness (per 100 µm)	295	51 (17%)	1.17 (0.93, 1.47)	0.26	1.16 (0.91, 1.47)	0.31
Gender	448	76 (17%)		0.39		0.51
Female	223	42 (19%)	Reference		Reference	
Male	225	34 (15%)	0.77 (0.42, 1.40)		0.82 (0.45, 1.50)	
Race	448	76 (17%)		**0.01**		**0.005**
White	193	28 (15%)	Reference		Reference	
African-American/More than one	213	30 (14%)	0.97 (0.48, 1.93)		0.997 (0.49, 2.01)	
Others/unknown	42	18 (43%)	4.42 (2.07, 9.43)		4.84 (2.36, 9.94)	
ROP stage	306	26 (8%)		0.93		0.95
<2	109	9 (8%)	Reference		Reference	
≥2	197	17 (9%)	1.05 (0.35, 3.16)		1.04 (0.35, 3.07)	
ROP treatment^†^	306	26 (43%)		0.89		0.89
No treatment	219	19 (9%)	Reference		Reference	
Treatment	50	7 (14%)	0.92 (0.27, 3.11)		0.92 (0.27, 3.10)	

GEE is used for accounting intereye correlation.

Boldface indicates statistical significance.

* Adjusted by PMA at imaging only.

† Treatment was after OCT session.

## Discussion

Few studies have compared comprehensive OCT measures and features between term and preterm infants, which is crucial for understanding retinal and choroidal development in preterm infants. The dearth of studies is partly due to the lack of universal screening, as term infants are typically not dilated for retinal examinations early after birth, in contrast to the routine ROP exams conducted on preterm infants. Using pooled OCT databases from multiple cohorts of infants in prior prospective studies, this study provides a comparison of retinal and choroidal measures as well as qualitative features in sizable cohorts of preterm and term infants at term-equivalent age. Our results revealed significant differences in retinal thickness measures, highlighting the increased inner retinal thickness at the fovea in preterm infants along with distinct qualitative features between preterm and term infants at term-equivalent age. These comprehensive analyses of qualitative and quantitative data suggest that by term-equivalent age, foveal inner retinal immaturity and photoreceptor underdevelopment are evident in preterm compared to term-born retinas.

Given that our study used multiple OCT databases from prior studies, inter- and intra-imager, as well as intra- and inter-device repeatability, was a critical consideration. The handheld OCT systems used in our study, compared to conventional tabletop systems, demonstrated good repeatability and reproducibility as evidenced by our previous study involving repeated measurements.^20^ We used the DOCTRAP software for our OCT platforms, validating it iteratively against manual segmentations by expert graders.^9^^,^^17^^,^^22^ Our earlier data indicate a coefficient of variation below 3.5% for central foveal thickness measurements across devices, confirming that our handheld OCT measurements and software analysis are reliable.^20^

The demographic and clinical characteristics of this study highlight significant differences between preterm and term infants (Table 1), indicating challenges in comparing OCT features and measures between these groups. In this population, the lack of significant differences in PMA at imaging between preterm and term infants, removed this confounding factor from the analysis. As expected, preterm infants exhibited significantly lower birth weight, which were strongly correlated with GA, as well as higher rates of systemic comorbidities. Racial distribution also varied significantly, with a higher proportion of African-American or multi-racial preterm infants. Previous research showed that foveal pit morphology differs by race and sex in adults, with males having thicker retinas than females and white populations having shallower foveal pits compared to African-Americans.^23^ However, these findings should be interpreted with caution, emphasizing that the observed anatomical differences may reflect the societal and economic influences on health rather than inherent genetic disparities, consistent with the understanding that race is a social construct.^24^ This underscored the need, in this study, to consider these variables when validating hypotheses regarding the effects of preterm birth, ROP, and choroidal thickness on retinal development. Accordingly, in our multivariate analyses, we used these factors, including GA (instead of birth weight because of collinearity), sex, race, ROP, and choroidal thickness, to assess their association with key OCT features and measures.

Our analyses revealed that preterm birth significantly impacts retinal development, particularly foveal maturation. Foveal pit development occurs predominantly during the last trimester of pregnancy, a critical period that can be disrupted in preterm infants.^3^ Previous research has demonstrated that the developing retina continues to undergo centrifugal inner retinal migration during 37 to 39 weeks PMA, leading to a thinner foveal center and deeper foveal pit, as observed through histology and OCT.^12^^,^^25^ This study found that preterm infants exhibited a notably altered foveal morphology (indicated by PF ratio of the total retina) at term-equivalent age, particularly an increased IRL thickness at the fovea (and thus reduced foveal pit depth), compared to that of term infants, aligning with the finding that extreme prematurity leads to these structural changes.^12^ Outer retinal thickness, in contrast, showed no significant difference at the fovea between preterm and term infants. The PF ratios of the total retina and IRLs were markedly different between preterm and term infants, whereas the PF ratio of the ORLs showed only slight differences between the groups (Table 3), suggesting that it is the differences in the IRLs that primarily drive the observed differences in the PF ratio of the total retina between preterm and term infants. However, among microstructural biomarkers of foveal outer retinal development, the presence of the foveal EZ band was significantly less frequent in preterm infants, with only 8.5% of preterms displaying a foveal EZ compared to 35.2% of full-term infants, suggesting a delay in photoreceptor development. Thus, the comprehensive thickness analyses of the total, inner, and ORLs at the fovea and parafovea and foveal microstructural biomarkers may indicate impaired or delayed centrifugal inner retinal migration and photoreceptor development during foveal maturation in preterm infants at term-equivalent age. Further longitudinal studies are needed to delineate the differences in the subsequent process of foveal differentiation between preterm and term infants, offering valuable insights into foveal maturation in preterm infants, particularly foveal hypoplasia seen in children with preterm birth.^26^

Based on the findings from the subgroup analyses, ROP appears to exert less impact on retinal development, particularly outer retinal development, in preterm infants compared to preterm birth itself. This study demonstrated no significant differences in foveal EZ and ELM between preterm infants with minimal and more severe stages of ROP. Although significant differences were observed in few qualitative OCT features between the two groups (Supplementary Fig. S1), multivariate analyses in Tables 4 and 5 revealed no significant associations between key qualitative/quantitative features and ROP severity (stage). Considering that infants with more severe ROP had a significantly lower GA and birth weight than those with minimal ROP, extreme prematurity may account for the significant differences in OCT features observed between preterm infants with minimal and more severe ROP in our study. This highlights the importance of addressing prematurity, especially extreme prematurity, to better understand foveal development and interpret OCT features in preterm infants at term-equivalent ages.

Several pathological features warrant further attention due to their marked differences between term and preterm infants. For example, IRF was predominantly observed in preterm infants (63.8%) compared to term infants (6.6%), whereas subfoveal SRF was exclusively identified in term infants. IRF, indicative of macular edema, has been reported as relatively common in preterm infants in previous OCT studies.^13^^,^^27^ The subfoveal SRF, observed in 7.0% of healthy term infants in this study, has been reported previously,^28^^,^^29^ although its prevalence and significance have not been thoroughly investigated. These findings suggest that these OCT features at term-equivalent age may serve as biomarkers distinguishing preterm and term birth, because a recent study also observed similar OCT features in both preterm and full-term infants, with CME in 22% of preterm infants and SRF only in full-term infants (15%).^30^ However, the underlying mechanisms behind these features, particularly the differences between term and preterm infants, remain unclear and warrant further investigation, including their long-term significance.

The choroid plays a crucial role in supplying oxygen and nutrients to the outer retina, including the fovea.^31^ Thus our study explored the relationship between choroidal thickness and retinal development, particularly outer retinal (photoreceptor) development. The results obtained indicate that preterm infants generally have reduced choroidal thickness compared with term infants. This reduction in choroidal thickness may be associated with delayed maturation of the foveal photoreceptor layer, as evidenced by the more frequent absence of the EZ and ELM lines in preterm infants (Table 3). However, the multivariate analysis did not show a significant association between choroidal thickness and outer retinal thickness (Table 4) or the foveal EZ band (Table 5). Instead, the foveal EZ band was significantly associated with PMA at imaging, suggesting that it may result from PMA-dependent maturation, independent of choroidal development. This seems to contrast with findings from a previous study, where photoreceptor development in term infants was dependent on choroidal development, with thinner foveal choroidal thickness associated with the absence of the foveal ellipsoid zone.^14^ However, in that study, the PMA at imaging was not accounted for, although imaging was done within 72 hours after birth in term infants, making direct comparison with our results from both preterm and term infants challenging.

Choroidal thickness was significantly associated with inner retinal thickness at the fovea, suggesting that a thinner choroid might be linked to the arrested or delayed maturation of the fovea due to impaired centrifugal inner retinal migration. Lawson et al.^32^ demonstrated that increased choroidal thickness is significantly associated with advanced foveal maturity—including decreasing inner retinal layers—suggesting that robust choroidal development contributes to proper foveal formation in both preterm and term infants, a finding that is compatible with our results. It is speculated that a thinner choroid may limit the availability of essential nutrients and oxygen to the fovea. This limitation could potentially interact with the process of inner retinal migration, which involves the centrifugal migration of retinal capillaries and the formation of the foveal avascular zone. It is hypothesized that centrifugal inner retinal migration relies on the development of the choroidal vasculature to ensure an adequate blood supply to the entire fovea as the development of these vascular systems is synchronized to meet the nutritional and oxygen requirements of the developing retina,^33^ suggesting the potential role of choroidal maturation in foveal development. However, our study cannot establish a causal association between delayed foveal development and a thinner choroid; instead, these may represent independent but correlated markers reflecting the impact of preterm birth on ocular development. This highlights the need for further research to clarify the role of choroid in foveal differentiation, particularly in the context of prematurity.

This study, while providing valuable insights into retinal development at term-equivalent age in preterm and term infants, has several limitations that should be considered. First, our study was cross-sectional in nature. Longitudinal studies, tracking preterm infants over time, are better suited to explore potential causal relationships or developmental trajectories. Particularly, whether our finding of arrested inner retinal displacement in preterm infants, compatible with the findings of O'Sullivan et al.,^12^ reflects a staggered delay or a slower or arrested trajectory requires further exploration through longitudinal studies in subsequent periods. Second, although the study population was relatively large, it was drawn from multiple databases with varying inclusion criteria and imaging protocols, which may have introduced variability in the data. Third, despite adjustments for several confounding variables, unmeasured factors such as environmental influences and systemic and CNS growth may affect retinal development and were not accounted for in the analysis. Another limitation is the potential impact of IRF on retinal thickness measurements. Although IRF can significantly influence retinal thicknesses, it was decided not to exclude cases with the fluid to avoid selection bias. Instead, INL, the most affected layer by the fluid, was separated from the inner retinal thickness measurements to minimize the confounding effect. However, this approach may not have fully addressed the complexities introduced by IRF. Another limitation of our study is the exclusion of 26 eyes (eight term and 18 preterm) because of indeterminate choroidal boundaries resulting from limited (choroidal) image quality. The proportion of eyes imaged with SS-OCT and the prevalence of limited image quality did not differ significantly between preterm and term infants. Although this exclusion represents a small fraction of the overall dataset, it may introduce selection bias and inherent differences between SD-OCT and SS-OCT could contribute to variability in choroidal measurements. Additionally, we acknowledge as a limitation that, without repeated measurements in this cohort, we could not directly assess intra- or inter-imager variability in thickness measurements; however, our previous study reported the excellent inter- and intra-imager repeatability and reproducibility of the handheld OCT system.^20^ Finally, the choriocapillaris was not investigated separately, as only overall choroidal thickness was used in this study, which may limit its ability to correlate with outer retinal development. These limitations highlight the need for further research to validate these findings and to explore the long-term implications of several retinal and choroidal alterations in preterm infants.

In conclusion, this comprehensive examination of retinal features and measures at term-equivalent age using handheld OCT highlights the profound impact of preterm birth on retinal maturation. These findings reveal significant differences in retinal thickness and qualitative features between preterm and term infants, particularly regarding increased foveal inner retinal thickness and certain developmental/pathological features in preterm infants. Although ROP influences certain pathological features, it appears to have only mild impact on retinal and choroidal OCT measures beyond that associated with extreme prematurity. Regarding the role of the choroid, while it may not play a significant part in overall retinal development, our data suggest that thinner choroid is associated with impaired inner retinal migration during foveal maturation. Future longitudinal studies are essential to further elucidate these relationships and to explore the long-term implications of these findings on foveal development and visual function in preterm infants.

## Supplementary Material

Supplement 1

Supplement 2
