# Supplementary material for: Comparison of Retinal and Choroidal OCT Measures and Features at Term Equivalent Age in Preterm and Term Infants
Source: Invest Ophthalmol Vis Sci. 2025 Oct 27;66(13):40. doi: 10.1167/iovs.66.13.40 (PMC12574739; doi:10.1167/iovs.66.13.40)
Supplement: Supplement 1 [file iovs-66-13-40_s001.pdf]

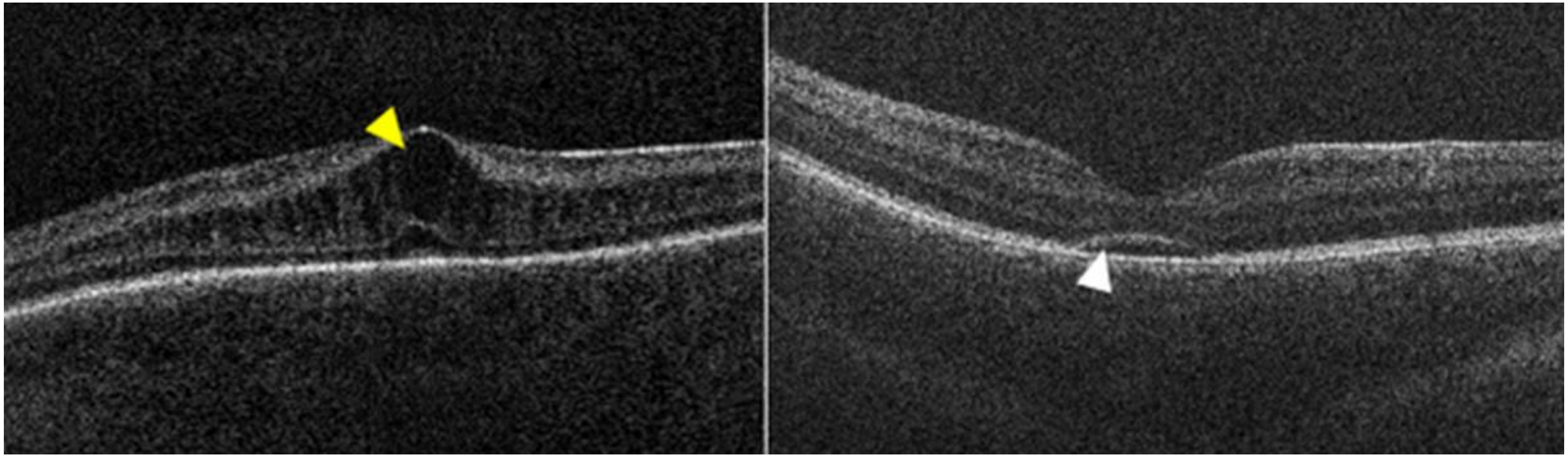

Supplementary Figure 1. Representative optical coherence tomography (OCT) images demonstrating qualitative features in preterm (left) and term (right) infants. Left panel: OCT from a preterm infant (born at 31+1 weeks' gestational age) shows a distinct pattern of intraretinal fluid (IRF), indicated by a yellow arrowhead. Right panel: OCT from a term infant reveals subretinal fluid (SRF), indicated by a white arrowhead.
