# Supplementary material for: Comparison of Retinal and Choroidal OCT Measures and Features at Term Equivalent Age in Preterm and Term Infants
Source: Invest Ophthalmol Vis Sci. 2025 Oct 27;66(13):40. doi: 10.1167/iovs.66.13.40 (PMC12574739; doi:10.1167/iovs.66.13.40)
Supplement: Supplement 2 [file iovs-66-13-40_s002.pdf]

**Supplementary Table 1.** Association of clinical characteristics and OCT features with inner retinal thickness at the fovea in preterm and full-term infants

| Variables | Inner retinal thickness at the fovea | | | | |
|---|---|---|---|---|---|
| | N (eyes) | Univariate | | Multivariate | |
| | | Regression coefficient | P-value | Regression coefficient | P-value* |
| **PMA at imaging** (per week) | 449 | -0.45 (1.24) | 0.72 | | |
| **GA at birth** (per week) | 449 | -1.96 (0.16) | **<0.001** | -1.96 (0.16) | **<0.001** |
| **Choroidal thickness** (per 100 μm) | 297 | -3.08 (1.08) | **0.005** | -3.02 (1.07) | **0.005** |
| | | **Mean (SE)** | | **Adjusted mean (SE)** | |
| **Gender** | 449 | | 0.19 | | 0.19 |
| Female | 224 (49.9%) | 38.54 (1.83) | | 38.57 (1.81) | |
| Male | 225 (50.1%) | 41.98 (1.86) | | 41.96 (1.86) | |
| **Race** | 449 | | **0.002** | | **0.002** |
| White | 193 (43.0%) | 44.88 (2.08) | | 44.90 (2.07) | |
| African-American/More than one | 213 (47.4%) | 38.39 (1.74) | | 38.36 (1.74) | |
| Others/unknown | 43 (9.6%) | 28.88 (3.70) | | 28.89 (3.68) | |
| **Foveal EZ band** | 447 | | **0.001** | | **0.001** |
| No | 371 (83.0%) | 42.21 (1.34) | | 42.25 (1.36) | |
| Yes | 76 (17.0%) | 30.33 (3.09) | | 30.15 (3.17) | |
| **ROP Stage** | 307 | | **<0.001** | | **<0.001** |
| <2 | 109 (35.5%) | 38.50 (1.91) | | 38.52 (1.91) | |
| ≥2 | 198 (64.5%) | 52.20 (1.93) | | 52.29 (1.93) | |
| **ROP treatment**[†] | 307 | | 0.17 | | 0.17 |
| No treatment | 220 (72%) | 45.28 (1.69) | | 45.29 (1.70) | |
| Laser photocoagulation | 50 (16%) | 49.65 (3.45) | | 49.70 (3.45) | |
| Anti-VEGF injection | 14 (5%) | 49.89 (8.86) | | 49.48 (8.90) | |
| Both | 23 (8%) | 60.47 (6.47) | | 60.48 (6.49) | |

GEE is used for accounting inter-eye correlation.
*Adjusted by PMA at imaging
[†]Treatment was after OCT session.

**Supplementary Table 2.** Association of clinical characteristics and OCT features with choroidal thickness over a central 2 mm across the fovea in preterm and full-term infants

| Variables | Choroidal thickness | | | | |
|---|---|---|---|---|---|
| | N (eyes) | Univariate | | Multivariate | |
| | | Regression coefficient | P-value | Regression coefficient | P-value* |
| **GA at birth** (per week) | 297 | 7.27 (1.65) | **<0.001** | 7.25 (1.65) | **<0.001** |
| | | **Mean (SE)** | | **Adjusted mean (SE)** | |
| **Gender** | 297 | | 0.26 | | 0.29 |
| Female | 147 (49.5%) | 302.12 (14.31) | | 301.40 (14.39) | |
| Male | 150 (50.5%) | 282.16 (10.17) | | 282.87 (10.08) | |
| **Race** | 297 | | 0.22 | | 0.22 |
| White | 127 (42.8%) | 286.50 (11.17) | | 285.76 (11.16) | |
| African-American/More than one | 144 (48.5%) | 280.21 (10.53) | | 280.51 (10.52) | |
| Others/unknown | 26 (8.8%) | 384.61 (54.00) | | 386.55 (54.31) | |
| **Foveal EZ band** | 295 | | 0.26 | | 0.30 |
| No | 244 (82.7%) | 288.43 (8.46) | | 288.86 (8.36) | |
| Yes | 51 (17.3%) | 314.13 (22.87) | | 312.09 (22.80) | |
| **ROP Stage** | 208 | | **0.001** | | **<0.001** |
| <2 | 79 (38.0%) | 301.71 (12.67) | | 303.03 (12.42) | |
| ≥2 | 129 (62.0%) | 246.01 (9.19) | | 245.21 (8.91) | |
| **ROP treatment**† | 208 | | 0.09 | | 0.09 |
| No treatment | 156 (75.0%) | 277.54 (8.73) | | 277.86 (8.58) | |
| Laser photocoagulation | 23 (11.1%) | 254.51 (19.08) | | 257.06 (17.85) | |
| Anti-VEGF injection | 8 (3.8%) | 285.03 (36.78) | | 270.02 (39.58) | |
| Both | 21 (10.1%) | 197.23 (25.47) | | 197.71 (24.30) | |

GEE is used for accounting inter-eye correlation.
*Adjusted by PMA at imaging
†Treatment was after OCT session.
